# Whole Genome Sequence-Based Identification of *Clostridium estertheticum* Complex Strains Supports the Need for Taxonomic Reclassification Within the Species *Clostridium estertheticum*

**DOI:** 10.3389/fmicb.2021.727022

**Published:** 2021-09-13

**Authors:** Joseph Wambui, Nicole Cernela, Marc J. A. Stevens, Roger Stephan

**Affiliations:** Institute for Food Safety and Hygiene, Vetsuisse Faculty, University of Zurich, Zurich, Switzerland

**Keywords:** *Clostridium estertheticum*, *Clostridium tagluense*, blown pack spoilage, vacuum packed meat, *rpoB*, core genome, pan genome

## Abstract

Isolates within the *Clostridium estertheticum* complex (CEC) have routinely been identified through the 16S rRNA sequence, but the high interspecies sequence similarity reduces the resolution necessary for species level identification and often results in ambiguous taxonomic classification. The current study identified CEC isolates from meat juice (MJS) and bovine fecal samples (BFS) and determined the phylogeny of species within the CEC through whole genome sequence (WGS)-based analyses. About 1,054 MJS were screened for CEC using quantitative real-time PCR (qPCR). Strains were isolated from 33 MJS and 34 BFS qPCR-positive samples, respectively. Pan- and core-genome phylogenomics were used to determine the species identity of the isolates. Average nucleotide identity (ANI) and digital DNA-DNA hybridization (dDDH) were used to validate the species identity. The phylogeny of species within the CEC was determined through a combination of these methods. Twenty-eight clostridia strains were isolated from MJS and BFS samples out of which 13 belonged to CEC. At 95% ANI and 70% dDDH thresholds for speciation, six CEC isolates were identified as genomospecies2 (*n*=3), *Clostridium tagluense* (*n*=2) and genomospecies3 (*n*=1). Lower thresholds of 94% ANI and 58% dDDH were required for the classification of seven CEC isolates into species *C. estertheticum* and prevent an overlap between species *C. estertheticum* and *Clostridium frigoriphilum*. Combination of the two species and abolishment of current subspecies classification within the species *C. estertheticum* are proposed. These data demonstrate the suitability of phylogenomics to identify CEC isolates and determine the phylogeny within CEC.

## Introduction

*Clostridium estertheticum* is a strict anaerobic spore forming psychrophile and the major cause of blown pack spoilage (BPS) of vacuum packed chilled meat (Collins, 1992; Kalchayanand et al., 1993). Based on an early 16S rRNA-based classification of genus *Clostridium*, *C. estertheticum* belongs to cluster I named *Clostridium sensu stricto* (Collins et al., 1994). Within this cluster, *C. estertheticum* constitute of a group of closely related psychrophilic *Clostridium* spp. [herein collectively referred to as *C. estertheticum* complex (CEC); after the species *C. estertheticum*, which was the first published member of the group] that are unable to grow above 28°C (Collins, 1992; Spring, 2003; Suetin et al., 2009). CEC constitutes of seven validly published species based on 16S rRNA classification.^1^ These include *C. algoriphilum*, *C. bowmanii*, *C. estertheticum*, *C. frigoris*, *C. lacusfryxellense*, *C. psychrophilum*, and *C. tagluense*, which were isolated from either terrestrial or marine sources (Collins, 1992; Spring, 2003; Shcherbakova et al., 2005; Suetin et al., 2009). Even though, *C. frigoriphilum* is not a validly published species, it is considered a species within the CEC (Pecheritsyna et al., 2007; Dorn-In et al., 2018). Two unnamed novel species within the CEC have also been proposed from recent studies (Brightwell and Horváth, 2018; Palevich et al., 2021). The majority of the CEC species have been linked with BPS or other types of meat spoilage (Húngaro et al., 2016).

Because of the relatedness of 16S rRNA gene sequences, the taxonomy and identification of CEC members has in most cases been debatable (Spring, 2003). This has resulted in ambiguous classification of identified strains as demonstrated in several studies, where strains have been identified as *C. estertheticum*-like, *C. frigoriphilum*-like, and *C. tagluense*-like (Brightwell and Clemens, 2012; Brightwell and Horváth, 2018; Dorn-In et al., 2018; Wambui et al., 2021). This is further exemplified by species *C. estertheticum*, whereby two strains *C. estertheticum* subp. *estertheticum* DSM 8809^T^ and *C. estertheticum* subp. *laramiense* DSM 14864^T^ were initially classified as two distinct species, *C. estertheticum* and *C. laramiense*, respectively (Collins, 1992; Kalchayanand et al., 1993). Consequent studies showed that the strains were genotypically closely related at the species level resulting into the combination of the two species into species *C. estertheticum* (Spring, 2003). Further extensive characterization of the two strains was carried out and it was concluded that even the subspecies classification was questionable (Yang et al., 2010). These data suggest that 16S rRNA may not offer the resolution needed to distinguish CEC at the species or subspecies level. This has implications on other 16S rRNA-based techniques such as restriction length fragment polymorphism (RLFP) that have previously been used for species level identification of CEC isolates (Broda et al., 2000; Cavill et al., 2011). Furthermore, it has direct implications on routine identification of presence of known causative agents of BPS such as *C. estertheticum* and closely related species within CEC, which are not known to cause BPS such as *C. lacusfryxellense* (Dorn-In et al., 2018).

Recently, whole genome sequencing (WGS) has become more common for bacterial identification purpose, thus facilitating the evaluation of the taxonomy of a group of interest in a phylogenetic context (Weigand et al., 2015). Furthermore, WGS has been proposed as a tool for revisiting the clostridial taxonomy beyond 16S rRNA-based classification so as to confirm monophyletic groups and/or redefine the groups taxonomically (Cruz-Morales et al., 2019). DNA-DNA hybridization (DDH) is one of the WGS-based experimental methods developed to determine the overall similarity between two genomes (Schildkraut et al., 1961; McCarthy and Bolton, 1963). Recently, an *in silico* based DDH, named digital DDH (dDDH) has been developed to complement the cumbersome DDH (Meier-Kolthoff et al., 2013). Both classical and digital DDH recommend values of >70% for bacteria intraspecies delineation (Schildkraut et al., 1961; McCarthy and Bolton, 1963; Meier-Kolthoff et al., 2013). Another *in silico* method used to determine similarity between two genomes is the average nucleotide identity (ANI; Kim et al., 2014). An ANI around 95–96% corresponds to the 70% DDH cut-off value for intraspecies delineation (Goris et al., 2007; Richter and Rosselló-Móra, 2009; Arahal, 2014).

Within the CEC, WGS-based analyses have so far been used in very few studies (Wambui et al., 2020d; Palevich et al., 2021). Furthermore, six species within the CEC lack published genomes hence the taxonomy of CEC beyond 16S rRNA-based classification is not fully known. Therefore, the aim of the current study was to identify newly isolated CEC strains from meat juice (MJS) and bovine fecal samples (BFS) using WGS. Furthermore, we aimed to resolve the phylogeny within the CEC on the basis of the genetic relatedness of the WGSs. By leveraging on 13 CEC strains identified presently, five newly sequenced genomes (representing five of the six unsequenced CEC species) and 16 publicly available CEC genomes, we have proposed a framework for the phylogeny of nine genomospecies within CEC through phylogenomics.

## Materials and Methods

### Sampling, Sample Handling, DNA Extraction, and qPCR Analysis

Over a period of 8months (April 2020–November 2020), 1,054 MJS were screened for the occurrence of *C. estertheticum* complex strains by quantitative real time-PCR (qPCR) as previously described with slight modifications (Wambui et al., 2020e). Samples were incubated anaerobically at 4°C for 2–3weeks. Current and subsequent anerobic incubations were carried out in rectangular anerobic box (7.0L; bioMérieux, Inc., Marcy l’Etoile, France) and the anaerobic conditions were generated by three 2.5L AnaeroGen Sachets (Thermo Fisher Scientific, Waltham, MA) per box. DNA extraction was carried out using the MagNa Pure LC DNA Isolation Kit III (Roche, Rotkreuz, Switzerland) by the MagNa Pure LC robotic workstation (Roche). A 100μl sample was mixed with 10μl of lysozyme (Sigma-Aldrich Chemie, Steinheim, Germany) to a concentration of 20mg/ml in phosphate-buffered saline (PBS; Sigma-Aldrich Chemie GmbH, Buchs, Switzerland) and incubated at 37°C for 30min. Subsequent steps from lysis with proteinase K to DNA elution was carried out according to the instruction manual of the DNA extraction kit. The primer and probes for *C. estertheticum* and closely related species (herein referred to as members of CEC) as well as the qPCR protocols used for the detection are as previously described (Brightwell and Clemens, 2012).

### *Clostridium* spp. Isolation and Presumptive Identification

Quantitative real time-PCR-positive MJS were further incubated anaerobically for 12weeks at 4°C. Isolation was carried out from the samples using two approaches. In the first approach, 14 samples were suspended (1:100) in pre-reduced Peptone Yeast Glucose Starch broth (PYGS; prepared in-house using the previously described formulation; Lund et al., 1993) and incubated anaerobically for 3–4weeks at 4°C followed by plating of a sample loopful on Columbia agar supplemented with 5% defibrinated sheep blood (CBA; Thermo Fisher Scientific, Waltham, MA). In the second approach, 19 samples were pre-treated in a multistep approach involving elimination of competitive microbiota and spore recovery, as previously described with slight modifications (Wambui et al., 2021). These steps were carried out aerobically. Briefly, 1ml of each sample was mixed with 1ml of absolute ethanol and incubated at 30°C for 1h. The mixture was then centrifuged at 12,000×*g* for 5min. The supernatant was discarded, and the resulting pellet resuspended in PBS containing lysozyme (4mg/ml) and incubated for 30min at 37°C. The prepared samples were serially diluted and plated on CBA and incubated anaerobically for 3–4weeks at 4°C. Finally, 34 qPCR-positive BFS from our previous study (Wambui et al., 2021) were also subjected to the second treatment procedure. The BFS had been incubated anaerobically for 24weeks at 4°C. Colonies with previously described characteristics of psychrophilic and psychrotrophic clostridia (Húngaro et al., 2016), were selected from each CBA plate showing growth and then purified twice anaerobically on CBA for 3weeks at 4°C prior to presumptive identification. All isolates were further identified using qPCR as described above and through matrix-assisted laser desorption-ionization time of flight mass spectrometry (MALDI-TOF; Bruker Biotyper system Version 3.0, Microflex LT/SH MS, Bruker Daltonics, Bremen, Germany) by using a-cyano-4-hydroxycinnamic acid as matrix. The system used FlexiControl and Biotyper real-time classification software (Bruker Daltonics). Further cultivation of the strains was carried out anaerobically on CBA at 8°C for 10–14days.

### Description of Reference *Clostridium* spp. Strains Used in the Current Study

Five type/representative strains; *C. bowmanii* DSM 14206^T^, *C. frigoriphilum* DSM 17811, *C. frigoris* DSM 14204^T^, *C. lacusfryxellense* DSM 14205^T^, and *C. psychrophilum* DSM 14207^T^, each representing a CEC species whose WGS has been lacking, were purchased from the Leibniz Institute DSMZ-German Collection of Microorganisms and Cell Cultures. Respective cultures were revived anaerobically in PYGS at 8°C for 2–3weeks. The strains were subsequently subjected to the presumptive identification tests mentioned above. Further cultivation of the strains was carried out anaerobically on CBA at 8°C for 10–14days.

### DNA Extraction and Whole Genome Sequencing

DNA extraction and WGS was performed as previously described (Wambui et al., 2020a). Briefly, the genomic DNA was isolated using the DNA blood and tissue kit (Qiagen, Hombrechtikon, Switzerland). The sequencing outputs, which were 150–300bp pair-ended reads, were prepared using Nextera DNA Flex chemistry using the Nextera DNA Flex Sample Preparation Kit (Ill) as per manfucaturer’s guidelines (Illumina, San Diego, CA, United States). The resulting transposon-based libraries were sequenced on a MiniSeq sequencer (Illumina) with a minimal coverage of 50×. The MiniSeq MidOutput Reagent Cartridge (300cycles) was used. Demultiplexing and adapter trimming was done using the Miniseq local run manager version 2.4.1 using standard settings. The reads were checked for quality using FastQC (Andrews, 2010) then assembled with SPAdes v. 3.12.0 (Bankevich et al., 2012) using Shovill 1.0.9.^2^ The quality of the genomes was checked using ContEST16S (Lee et al., 2017) and CheckM (Parks et al., 2015). We were unable to revive *C. frigoriphilum* DSM 17811 and *C. psychrophilum* DSM 14207^T^ cultures, hence their DNA samples were purchased from the Leibniz Institute DSMZ and sequenced as described above. The complete 16S rRNA sequences were extracted from the WGS of the 27 isolated strains (only an incomplete sequence was extracted for strain CM008, hence it was omitted from the analysis) and 32 representative clostridia strains using ContEST16S (Lee et al., 2017). The sequences were aligned in CLC Workbench Genomics v. 8.1 (Qiagen, Aarhus, Denmark) using the progressive alignment algorithm with default settings whereby, the gap open cost, gap extension cost, and end gap cost were set to 10.0, 1.0 and as any other, respectively. The alignment process was set to very accurate (slow). The 16S rRNA phylogenetic tree was created from the aligned sequences in the CLC Workbench Genomics using the Maximum likelihood Phylogeny method. The tree construction method and nucleotide substitution model were set to neighborhood joining method and Jukes Cantor model, respectively, while the transition/transversion ratio was set to 2.0. Bootstraps were based on 1,000 replicates.

### Whole Genome Sequence-Based *in silico* Strain Identification and Phylogenomic Analysis

Pan- and core genome-based phylogenomic trees were created from the WGS of the 28 isolates from this study (their accession numbers are given in Supplementary Table 1), five newly sequenced genomes each representing an unsequenced CEC species (Supplementary Table 2) and 27 strains either from our previous studies (Wambui et al., 2020a,b,c,d, 2021) or deposited in NCBI database,^3^ respectively. Besides CEC, the other representative clostridia groups were *C. algidicarnis*, *C. gasigenes*, and *C. frigidicarnis* that are usually isolated from meat and/or associated with BPS of vacuum-packed chilled meat (Húngaro et al., 2016) as well as *C. argentinense* and *C. peptidovorans* that are closely related to CEC (Lawson and Rainey, 2016). The list of the genomes of the 27 reference/presentative clostridia strains including their accession numbers are given in Supplementary Tables 3 and 4.

The core and pan genomes trees were constructed as previously described (Stevens et al., 2019) using the Bacterial Pan Genome Analysis (BPGA) software package with a 70% sequence identity cut-off (Chaudhari et al., 2016). Trees were constructed in BPGA using standard settings and the resulting newick files visualized in iTOL (Letunic and Bork, 2019). Pairwise nucleotide comparisons (ANI and digital dDDH) were determined *in silico* for the 60 WGS to determine the correct species assignment of the CEC isolates. The dDDH was carried out using digital DMSZ’s Genome-to-Genome Distance Calculator web server (Meier-Kolthoff et al., 2013). The ANI was determined with pyANI (Pritchard et al., 2016) using the BLAST algorithm.

### Complete Genome Sequencing of Strain CF002 and Targeted Gene Sequence Analyses for Strains CF002, *C. estertheticum* DSM 8809^T^ and *C. frigoriphilum* DSM 17811

Targeted gene analyses were carried out for further determination of the correct species assignment of *C. frigoriphilum* DSM 17811. Strain CF002 was first subjected to long-read (MinION, Oxford Nanopore Technologies) sequencing. In brief, genomic DNA was extracted using the MasterPure Complete DNA and RNA Purification Kit (Lucigen LubioScience, Zürich, Switzerland). Multiplex libraries were prepared using the SQK-LSK109 ligation sequencing kit with the EXP-NBD104 native barcoding expansion kit (ONT, Oxford, United Kingdom) and sequenced on a MinION Mk1B device using the FLO-MIN106 (R9) flow cell (ONT). Hybrid assemblies were generated using Unicycler v0.4.8 (Wick et al., 2017) with default settings. Secondly, all 16S rRNA sequences of strains CF002 and *C. estertheticum* DSM 8809^T^ were extracted *in silico* then compared through sequence alignment with the NCBI-catalogued and *in silico* extracted 16S rRNA sequences of *C. frigoriphilum* DSM 17811. The *in silico* extraction of 16S rRNA sequences was carried out using ContEST16S (Lee et al., 2017). To further validate the correct assignment of DSM 17811, the *rpoB* gene sequences were identified in the 60 genomes using blast*n* and extracted from the genome with samtools using the faidx option and the blast results as input (Altschul et al., 1990; Li et al., 2009). The sequences were aligned and the *rpoB* phylogenetic tree was created in CLC Workbench Genomics as described above.

## Results

### Isolation and Presumptive Identification of CEC Strains

A total of 28 presumptive clostridia isolates (21 from MJS and seven from BFS) were isolated (Table 1). Using qPCR, 13 out of the 28 isolates were identified as members of CEC. This was further collaborated by the 16S rRNA-based phylogenetic tree (Supplementary Figure 1). Further analysis of the isolates with MALDI-TOF identified 10 isolates of the 28 isolates as *C. algidicarnis*.

**Table 1:** Presumptive identification of *Clostridium estertheticum* complex (CEC) and other *Clostridium* spp. strains isolated from meat juice samples (MJS) and bovine fecal samples (BFS).

Strain ID	Sample	qPCR^*^	MALDI-TOF
CM005	MJS	–	–
CM006	MJS	–	*C. algidicarnis*
CM007	MJS	–	–
CM008	MJS	CEC	–
CM009	MJS	–	*C. algidicarnis*
CM010	MJS	–	*C. algidicarnis*
CM011	MJS	–	*C. algidicarnis*
CM012	MJS	–	*C. algidicarnis*
CM013	MJS	–	*C. algidicarnis*
CM014	MJS	–	–
CM015	MJS	–	*C. algidicarnis*
CM016	MJS	–	–
CM017	MJS	–	*C. algidicarnis*
CM018	MJS	CEC	–
CM020	MJS	CEC	–
CM024	MJS	CEC	–
CM026	MJS	–	–
CM027	MJS	CEC	–
CM028	MJS	CEC	–
CM029	MJS	–	*C. algidicarnis*
CM030	MJS	–	*C. algidicarnis*
CF001	BFS	CEC	–
CF002	BFS	CEC	–
CF008	BFS	CEC	–
CF009	BFS	CEC	–
CF011	BFS	CEC	–
CF012	BFS	CEC	–
CF013	BFS	CEC	–

* Quantitative real-time PCR (qPCR) developed by Brightwell and Clemens (2012) to detect CEC species; *Clostridium bowmanii*, *Clostridium estertheticum*, *Clostridium frigoris*, *Clostridium frigoriphilum*, *Clostridium lacusfryxellense*, *Clostridium psychrophilum*, *Clostridium tagluense*, and other related CEC species. The dash (–) indicates the isolate was negative for CEC in the qPCR assay or unidentifiable by matrix-assisted laser desorption-ionization time of flight mass spectrometry (MALDI-TOF).

### Whole Genome Sequence-Based Phylogenomics Increases the Resolution for Identifying and Discriminating Species Within the *Clostridium estertheticum* Complex

**Figure 1:**
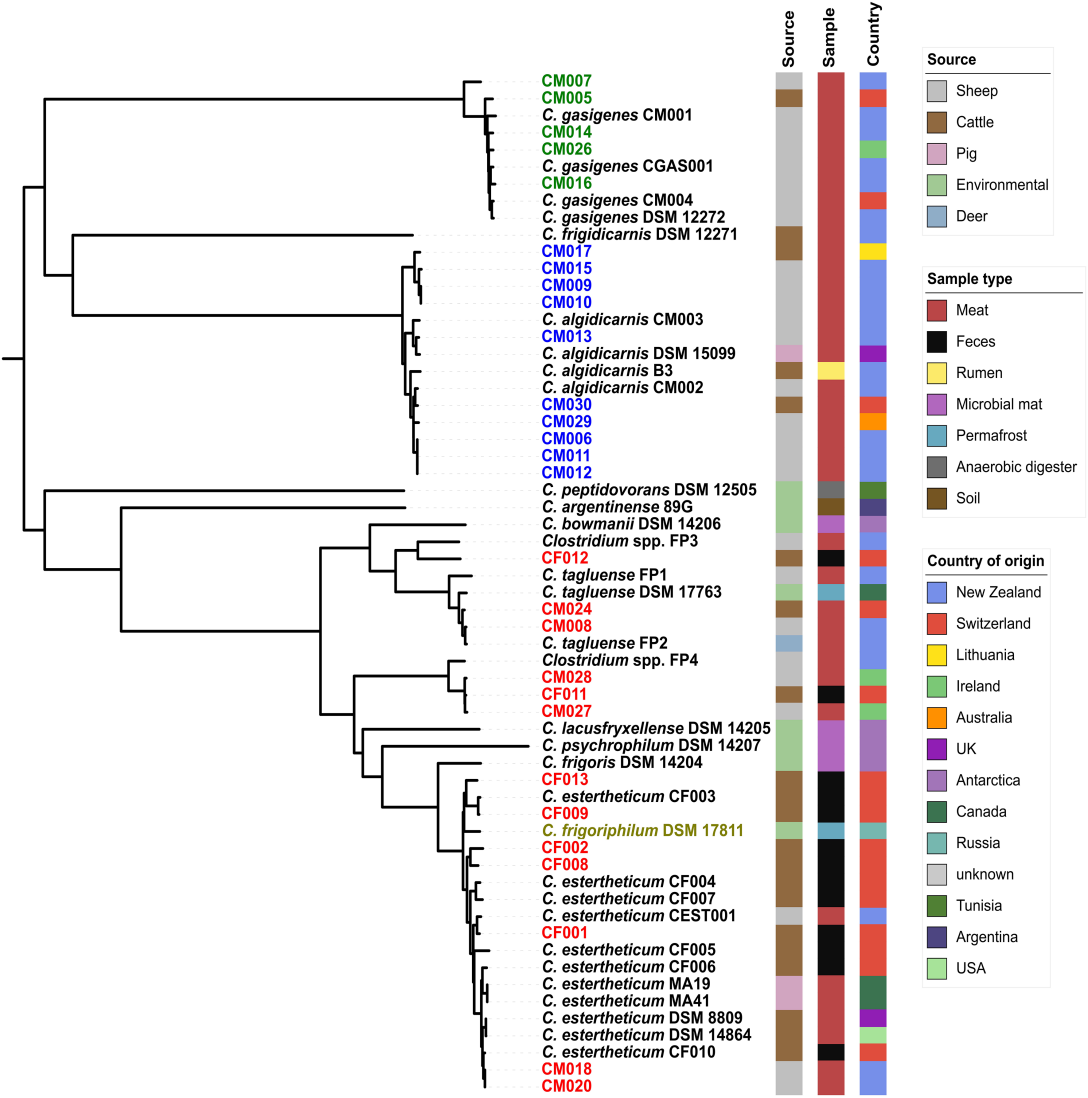
Core-genome phylogenomic tree of 28 *Clostridium* spp. strains isolated from meat juice (MJS) and bovine fecal samples (BFS) and 32 representative clostridia strains. Thirteen isolates (red) clustered within the *Clostridium estertheticum* complex (CEC). Other isolates clustered within species *C. algidicarnis* (blue text; *n*=10) and *C. gasigenes* (green text; *n*=5). *C. frigoriphilum* DSM 17811 is highlighted in olive. On the right of the tree are metadata of the 60 clostridia strains.

**Figure 2:**
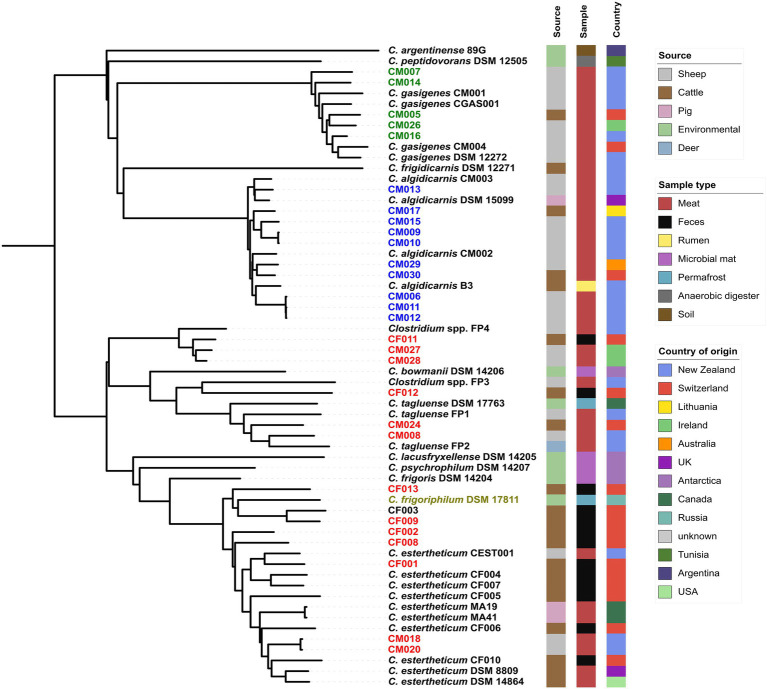
Pan-genome phylogenomic tree of 28 *Clostridium* spp. strains isolated from MJS and BFS and 32 representative clostridia strains. Thirteen isolates (red) clustered within the CEC. Other isolates clustered within species *C. algidicarnis* (blue text; *n*=10) and *C. gasigenes* (green text; *n*=5). *C. frigoriphilum* DSM 17811 is highlighted in olive. On the right of the tree are metadata of the 60 clostridia strains.

We created pan-genome (Figure 1) then core-genome (Figure 2) based phylogenomic trees for the 28 isolates from this study and 32 clostridia representative strains. From the phylogenomic trees, three CEC isolates from this study (CF011, CM027, and CM028) formed a monophyletic clade with *Clostridium* spp. FP4 indicating that all four strains constituted a single species; herein referred to as genomspecies2. Two CEC isolates, CM008 and CM028, formed a monophyletic clade with *C. tagluense* representative strains suggesting they belong to species *C. tagluense*. Furthermore, CF012 formed a monophyletic clade with *Clostridium* spp. FP3, suggesting that it either belonged to genomospecies1 or a novel species. All other seven strains formed a monophyletic clade with *C. estertheticum* representative strains hence suggesting they belong to species *C. estertheticum*. A surprising result from the phylogenomic trees was the monophyletic clade between species *C. estertheticum* and *C. frigoriphilum* (Figures 1, 2), which suggested that they constitute a single species. This was further collaborated by extracted 16S rRNA complete sequences of *C. estertheticum* DSM 8809^T^ (*n*=16) and strain CF002 (*n*=17). One variant of CF002 was identical with the complete *in silico* extracted (*n*=1) and the nearly complete publicly available (*n*=1) 16S rRNA sequences of *C. frigoriphilum* DSM 17811. Another sequence variant of CF002 was identified in DSM 8809^T^ (Supplementary Figure 2). This suggested species *C. estertheticum* exhibits intragenomic and intergenomic 16S rRNA heterogeneity that might have led to prior classification of *C. estertheticum* and *C. frigoriphilum* as two distinct species. Further proof for the relatedness of species *C. estertheticum* and *C. frigoriphilum* was observed in the *rpoB* gene-based phylogeny of all 60 clostridia genomes (Supplementary Figure 3), where *C. frigoriphilum* DSM 17811 was within a monophyletic clade dominated by *C. estertheticum* representative strains.

### Whole-Genome Alignment (ANI and dDDH) Data Supports Phylogenomic-Based Identification of Species Within *Clostridium estertheticum* Complex

**Figure 3:**
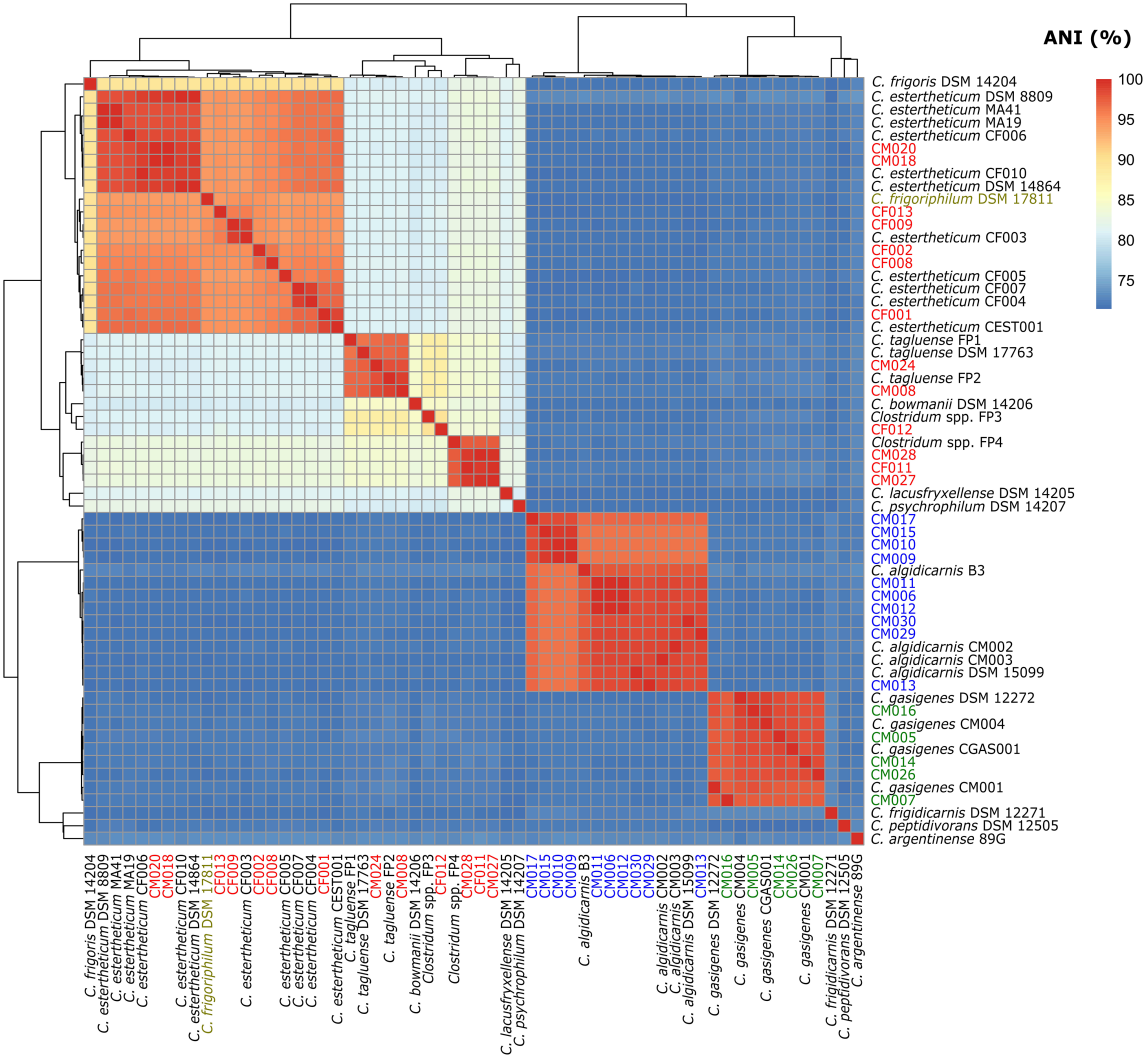
Heat map of average nucleotide identity (ANI) analysis of 28 *Clostridium* spp. strains isolated from MJS and BFS and 32 representative clostridia strains. Thirteen isolates belonging to the *Clostridium estertheticum* complex are highlighted in red. Other isolates within species *C. algidicarnis* (blue text; *n*=10) and *C. gasigenes* (green text; *n*=5) are also highlighted. *C. frigoriphilum* DSM 17811 is highlighted in olive.

**Figure 4:**
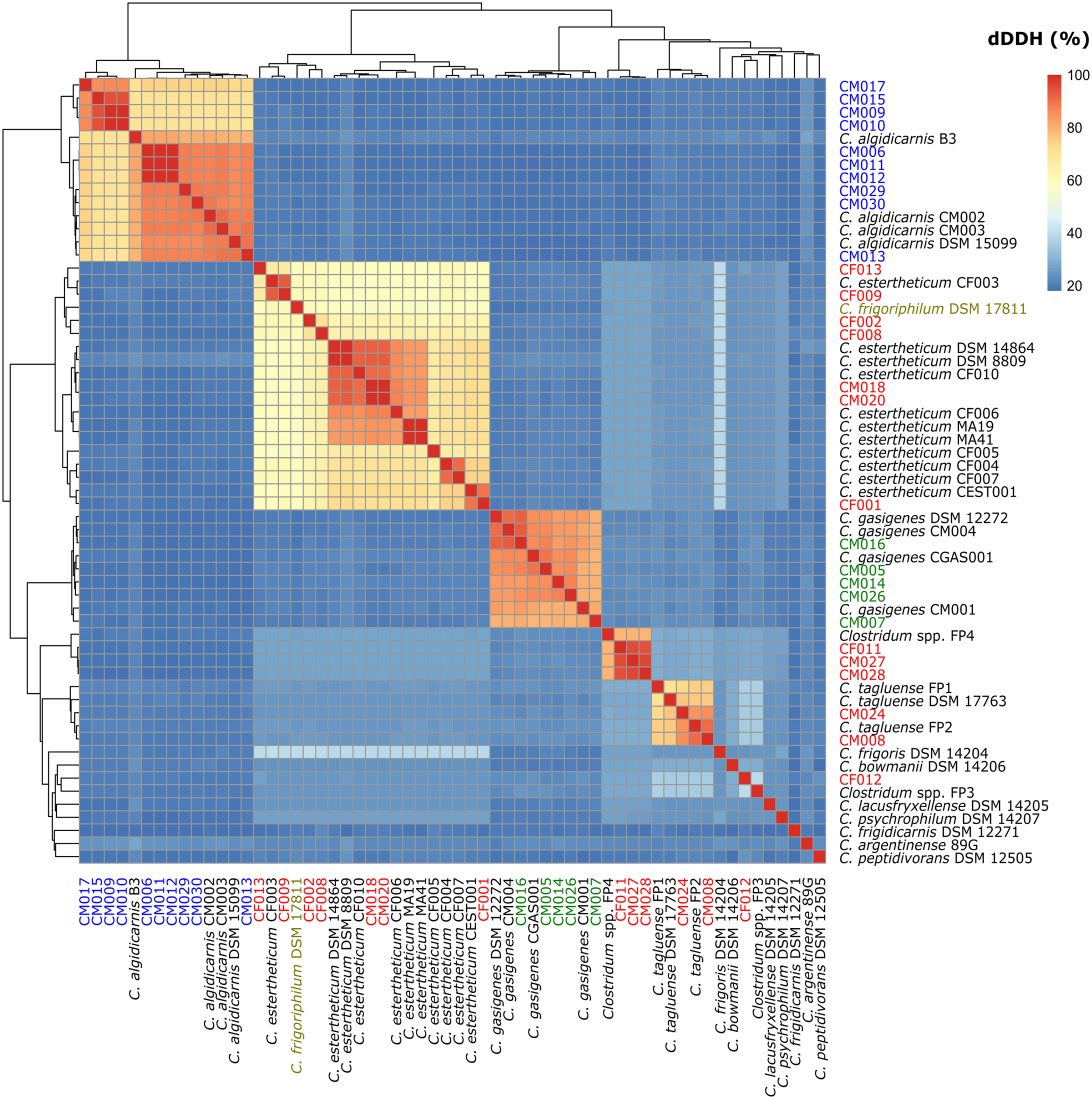
Heat map of digital DNA-DNA hybridization (dDDH) analysis of 28 *Clostridium* spp. strains isolated from MJS and BFS and 32 representative clostridia strains. Thirteen isolates belonging to the *Clostridium estertheticum* complex are highlighted in red. Other isolates within species *C. algidicarnis* (blue text; *n*=10) and *C. gasigenes* (green text; *n*=5) are also highlighted. *C. frigoriphilum* DSM 17811 is highlighted in olive.

To confirm the species identification of the MJS and BFS isolates and define overall interspecies relatedness within the CEC, we calculated the pairwise nucleotide-level comparisons [ANI (Figure 3) and dDDH (Figure 4)] using the WGS of the 60 strains. Our focus was first on the six CEC strains belonging to three different species, but closely related to species *C. tagluense*. The ANI and dDDH values between genomospecies2 representative strain FP4 and the strains CF011, CM027, and CM028 ranged between 97.47–97.48 and 78.7–78.9%, respectively. On the other hand, the ANI and dDDH values among strains CM008 and CM024 and *C. tagluense* representative strains were 96.88–98.89 and 73.6–90.7%, respectively. These values were above the ANI and dDDH threshold for intraspecies delineation confirming the correct species assignment. Finally, ANI and dDDH values of strain CF012 with genomospecies1 representative strain FP3, its closest related strain, were 89.70 and 40.0%, respectively, confirming that strain CF012 was a novel species; herein referred to as genomospecies3. We further shifted our focus to the seven strains from this study identified as *C. estertheticum* and lastly to *C. frigoriphilum* DSM 17811. First, we noted that ANI values were 94.27–94.61% when strains CF009 and CF013 were compared with *C. estertheticum* DSM 14864^T^ and DSM 8809^T^. On the other hand, the dDDH values were 58.5–68.0% when strains CF005, CF008, CF009, and CF013 were compared with the DSM 14864^T^ and DSM 8809^T^. Comparing *C. frigoriphilum* DSM 17811 with the DSM 14864^T^ and DSM 8809^T^ showed the ANI and dDDH values were 94.41–94.50 and 58.2–58.5%, respectively. Comparatively, these values were below the 95.0% ANI and 70% dDDH threshold for speciation, which suggested that using these values as a cut-off for the classification of species *C. estertheticum* and *C. frigoriphilum* would cause an overlap between the two species *C. estertheticum*.

### ANI and dDDH Thresholds of ≈94.0 and ≈58.0%, Respectively, Harmonize the Taxonomic Overlap Between the *Clostridium estertheticum* and *C. frigoriphilum* Genomospecies

In order to harmonize taxonomic inconsistences between species *C. estertheticum* and *C. frigoriphilum*, lower ANI and dDDH thresholds that deviates from the canonical thresholds were required. In this case, we noticed that using 94.0% ANI and 58.0% dDDH as the cut-off eliminates the overlap arising from existing taxonomy and encompasses all strains within the two species in a single monophyletic clade. The values provide a basis for combining species *C. estertheticum* and *C. frigoriphilum*.

### cgMLST, ANI, and dDDH Refutes the Classification of *C. estertheticum* Genomospecies into *C. estertheticum* Subspecies *estertheticum* and *C. estertheticum* Subspecies *laramiense*

Finally, we investigated the validity of the classification of *C. estertheticum* into *C. estertheticum* subsp. *estertheticum* and *C. estertheticum* subsp. *laramiense*. In this case, evaluation of the core and pangenome trees showed that the two *C. estertheticum* type strains DSM 8809^T^ and DSM 14864^T^, which are the type strains for *C. estertheticum* subsp. *estertheticum* and *C. estertheticum* subsp. *laramiense*, respectively, clustered together. Furthermore, pairwise comparison of ANI and dDDH values within the *C. estertheticum* genomospecies, showed that at ANI 99.8% and dDDH 99.2%, the two strains were more closely related to each other than any other strain. The close relationship was also evident in the *rpoB*-based phylogeny (Supplementary Figure 3). These data provide a basis to contest the current subspecies within the species *C. estertheticum*.

## Discussion

*C. estertheticum* is the major causative agent for BPS of vacuum-packed chilled meat and the occurrence of BPS is associated with high economic losses for the global meat industry (Wambui and Stephan, 2019). A qPCR developed to detect indiscriminatory *C. estertheticum* and related species (Brightwell and Clemens, 2012), herein, referred collectively as CEC, showed 3.1% of 1,054 MJS were contaminated with CEC species. Given that we used a non-discriminating qPCR protocol, we used WGS methods to identify the strains to species level. Using pan-genome and core-genome based phylogenomics (Figures 1, 2), we could identify the CEC isolates from MJS and BFS. Specifically, six strains could be identified as belonging to *C. tagluense*, genomospecies2 and novel genomospecies3. Similarly, seven strains could be identified as belonging to species *C. estertheticum*.

Whole genome sequence-based analyses reveal evolutionary relatedness of species allowing for taxonomic harmonization of species that are nearly indistinguishable by their 16S rRNA sequences (Carroll et al., 2020). This was clearly demonstrated in our current analysis whereby the newly sequenced *C. frigoriphilum* DSM 17811 formed a monophyletic clade with *C. estertheticum* reference strains. Despite being a non-validly published species, *C. frigoriphilum* has been considered a distinct species within the CEC (Mang et al., 2021). However, our data highly suggest that *C. frigoriphilum* is not an own species but belongs to species *C. estertheticum*. It is therefore unsurprising that strains previously identified as *C. frigoriphilum* or *C. frigoriphilum*-like have been shown to cause BPS with corresponding production of high levels of gas (Dorn-In et al., 2018; Mang et al., 2021), which is characteristic of the species *C. estertheticum* (Zhang et al., 2019). Furthermore, strains previously identified as *C. frigoriphilum* or *C. frigoriphilum*-like isolates have been noted to be either hemolytic or non-hemolytic (Mang et al., 2021), which is also a characteristic of species *C. estertheticum* (Spring, 2003; Yang et al., 2010; Esteves et al., 2020, 2021). Finally, the MALDI-TOF spectra of *C. frigoriphilum* or *C. frigoriphilum*-like strains were shown to be nearly identical to those of *C. estertheticum* strains (Dorn-In et al., 2018; Mang et al., 2021). Through complete genome sequencing of strain CF002, we demonstrated that on one hand, the strain shares a similar variant of the 16S rRNA with the catalogued sequence of strain DSM 17811, and on the hand, shares another variant with *C. estertheticum* DSM 8809^T^. While we could not completely sequence DSM 17811, we have demonstrated that intragenomic 16S rRNA heterogeneity within species *C. estertheticum* might be rampant. We have also provided further evidence that species *C. estertheticum* has among the highest number of 16S rRNA copies (*n*=16–17 for DSM 8809^T^ and CF002, respectively; Supplementary Figure 2) among known bacteria species.^4^ The multiplicity and variability of 16S rRNA within the species *C. estertheticum* may have contributed to misclassification of *C. frigoriphilum* as a distinct species. We identified that the *rpoB* gene also resulted in the accurate assignment of isolates to specific species (Supplementary Figure 3). The gene avoids limitations inherent in 16S rRNA gene intraspecies heterogeneity (Dahllof et al., 2000; Ogier et al., 2019). The *rpoB*-based phylogeny not only identified the MJS and BFS sample isolates in a manner that replicated the core- and pangenome analysis, but also provided further evolutionary proof that species *C. frigoriphilum* and *C. estertheticum* are related. The *rpoB* gene therefore is a promising marker for developing future rapid and accurate detection and/or quantification assays for CEC.

Comparative studies between ANI and DDH values show that ANI values of 95–96% are equivalent to the 70% DDH (Goris et al., 2007; Richter and Rosselló-Móra, 2009). In this regard, a recent study used ANI of 96% to delineate six strains into distinct CEC species and showed the value was sufficient (Palevich et al., 2021). In the present study, we broadened the analysis to encompass 34 CEC genomes, including five reference strains whose genomes were lacking. Similar to the previous study (Palevich et al., 2021), the 96% ANI was sufficient to delineate all CEC species apart from *C. estertheticum*. Comparatively, the 70% dDDH for intraspecies delineation (Meier-Kolthoff et al., 2013) also sufficed for all genomospecies apart from *C. estertheticum*. We observed that lower thresholds of 58% dDDH and 94% ANI were required to encompass all strains within the *C. estertheticum* monophyletic clade. While most bacterial species fall within the 95–96% ANI range (Jain et al., 2018), it is not uncommon for bacterial species to exhibit lower intraspecies ANI values. A threshold of 94% ANI was also proposed as the putative boundary for intraspecies delineation and was shown to work excellently in mirroring a lower DDH range of ≈60–70% (Richter and Rosselló-Móra, 2009), which is consistent with our values for species *C. estertheticum*. Given the other CEC species were represented by one to five genomes, the revelation that lower dDDH and ANI values were required for species *C. estertheticum*, the application of 70% dDDH and 95–96% ANI thresholds for intraspecies delineation within CEC have to be used with caution in future studies.

Based on previous studies, the classification of species *C. estertheticum* into *C. estertheticum* subsp. *estertheticum* and *C. estertheticum* subsp. *laramiense* has been an ongoing debate (Spring, 2003; Yang et al., 2010). Using WGS-based analyses, which consisted of dDDH and ANI, core- and pan-genome phylogenomics and *rpoB* phylogenetic evaluation of DSM 8809^T^ and DSM 14864^T^, we have evidentiary shown that the two strains are indeed closely related at the genomic level. The data strongly supports the previous study (Yang et al., 2010) that called into question the current classification of *C. estertheticum* species into *C. estertheticum* subsp. *estertheticum* and *C. estertheticum* subsp. *laramiense*.

As previously stated, an ideal taxonomy should be interpretable and provide the required resolution for species delineation (Carroll et al., 2020). In this regard, we propose a taxonomic framework for CEC members consisting of validly published, novel and amended species. Based on WGS-based analyses, we have confirmed the validity of five published CEC species; *C. bowmanii*, *C. frigoris*, *C. lacusfryxellense*, *C. tagluense*, and *C. psychrophilum*. In addition, we have validated a previous report that strains *Clostridium* spp. FP3 and *Clostridium* spp. FP4 represent novel CEC genomospecies (Palevich et al., 2021) and we propose the novel species be identified as genomospecies1 and genomospecies2, respectively. CF012, which was isolated in the present study from BFS has also been established to constitute a novel species, which we propose it be identified as genomospecies3. Furthermore, we propose two amendments of species *C. estertheticum*. The first amendment is to combine the previous taxonomic ranks, *C. frigoriphilum* and *C. estertheticum* into *C. estertheticum*. Secondly, we propose the removal of the subspecies classification of *C. estertheticum* until suitable and sufficient phenotypic and genotypic data are available to warrant such classification. Although, we have identified nine CEC genomospecies, it is important to recognize that *C. algoriphilum* is also considered a member of the CEC (Shcherbakova et al., 2005; Yang et al., 2014). Its exclusion in the current analyses was as result of unavailability of its genome and further studies will be required to validate its taxonomic status.

## Conclusion

We have used WGS-based methods, including pan- and core-genome phylogenomics and pairwise nucleotide-level comparisons (dDDH and ANI), to identify 13 MJS and BFS CEC isolates to the species level and determine the phylogeny of CEC species. The 13 isolates belonged to species *C. estertheticum* (*n*=7), genomospecies2 (*n*=3), *C. tagluense* (*n*=2), and genomospecies3 (*n*=1). These results were replicated by *rpoB* gene-based phylogeny. By leveraging on the genomes of the 13 CEC isolates, newly sequenced genomes of five CEC species and 16 publicly available CEC genomes, we identified nine distinct species within CEC through the phylogenomics. These include validly published species; *C. bowmanii*, *C. estertheticum*, *C. frigoris*, *C. lacusfryxellense*, *C. tagluense*, and *C. psychrophilum* and novel species; genomospecies1, genomospecies2, and genomospecies3. More importantly, the analyses have shown species *C. estertheticum* and *C. frigoriphilum* are related which has led to the formulation of parameters that harmonize the taxonomic classification of species *C. estertheticum*. Specifically, we have proposed that 58% dDDH and 94% ANI be applied for delineating species *C. estertheticum* to include strains previously identified as species *C. frigoriphilum*. The WGS-based analyses have also provided a basis to propose the abolishment of the current subspecies classification within species *C. estertheticum*. Taken together, we have shown that WGS-based methods are suitable for identifying CEC isolates to species level and determining the phylogeny of CEC species.

## Data Availability Statement

The datasets presented in this study can be found in online repositories. The names of the repository/repositories and accession number(s) can be found in the article/Supplementary Material.

## Author Contributions

JW and RS designed the study. JW performed the experiments and wrote the initial draft manuscript. JW, MS, and NC performed whole genome sequencing and bio-informatic analyses. JW, RS, and MS revised the final manuscript. RS supervised the study. All authors contributed to the article and approved the submitted version.

## Conflict of Interest

The authors declare that the research was conducted in the absence of any commercial or financial relationships that could be construed as a potential conflict of interest.

## Publisher’s Note

All claims expressed in this article are solely those of the authors and do not necessarily represent those of their affiliated organizations, or those of the publisher, the editors and the reviewers. Any product that may be evaluated in this article, or claim that may be made by its manufacturer, is not guaranteed or endorsed by the publisher.

## Footnotes

^1^www.bacterio.net/clostridium.html (Accessed June 2021).

^2^ https://github.com/tseemann/shovill

^3^ https://www.ncbi.nlm.nih.gov

^4^ https://rrndb.umms.med.umich.edu/
