# Supplementary material for: Whole Genome Sequence-Based Identification of Clostridium estertheticum Complex Strains Supports the Need for Taxonomic Reclassification Within the Species Clostridium estertheticum
Source: Front Microbiol. 2021 Sep 13;12:727022. doi: 10.3389/fmicb.2021.727022 (PMC8473909; doi:10.3389/fmicb.2021.727022)
Supplement: Supplementary file 1 [file Data_Sheet_1.docx]

Supplementary Material


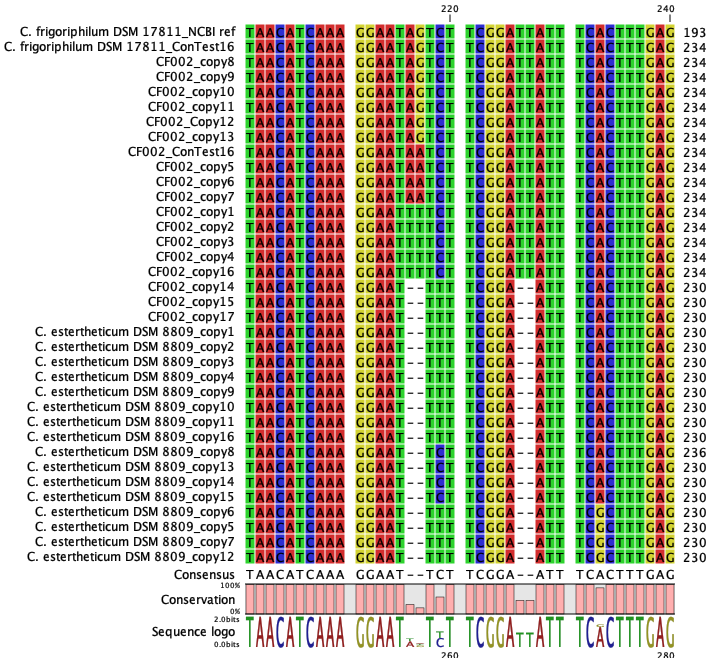


A

B

**Supplementary Figure 1.** 16S rRNA sequence alignment of *Clostridium frigoriphilum* DSM 17811, *C. estertheticum* DSM 8809^T^ and strain CF002. The region used to develop probes to differentiate species *C. frigoriphilum* and *C. estertheticum* is highlighted in black. Both sequences of *C. frigoriphilum* DSM 17811 that were either downloaded from NCBI database or extracted *in silico* through ConTest16 were similar to one of strain CF002’s 16S rRNA variants (Arrow A). A second 16S rRNA allele of CF002 was also similar to one of *C. estertheticum* DSM 8809^T^’s 16S rRNA variant (Arrow B). These data further support both species *C. frigoriphilum* and *C. estertheticum* are related.


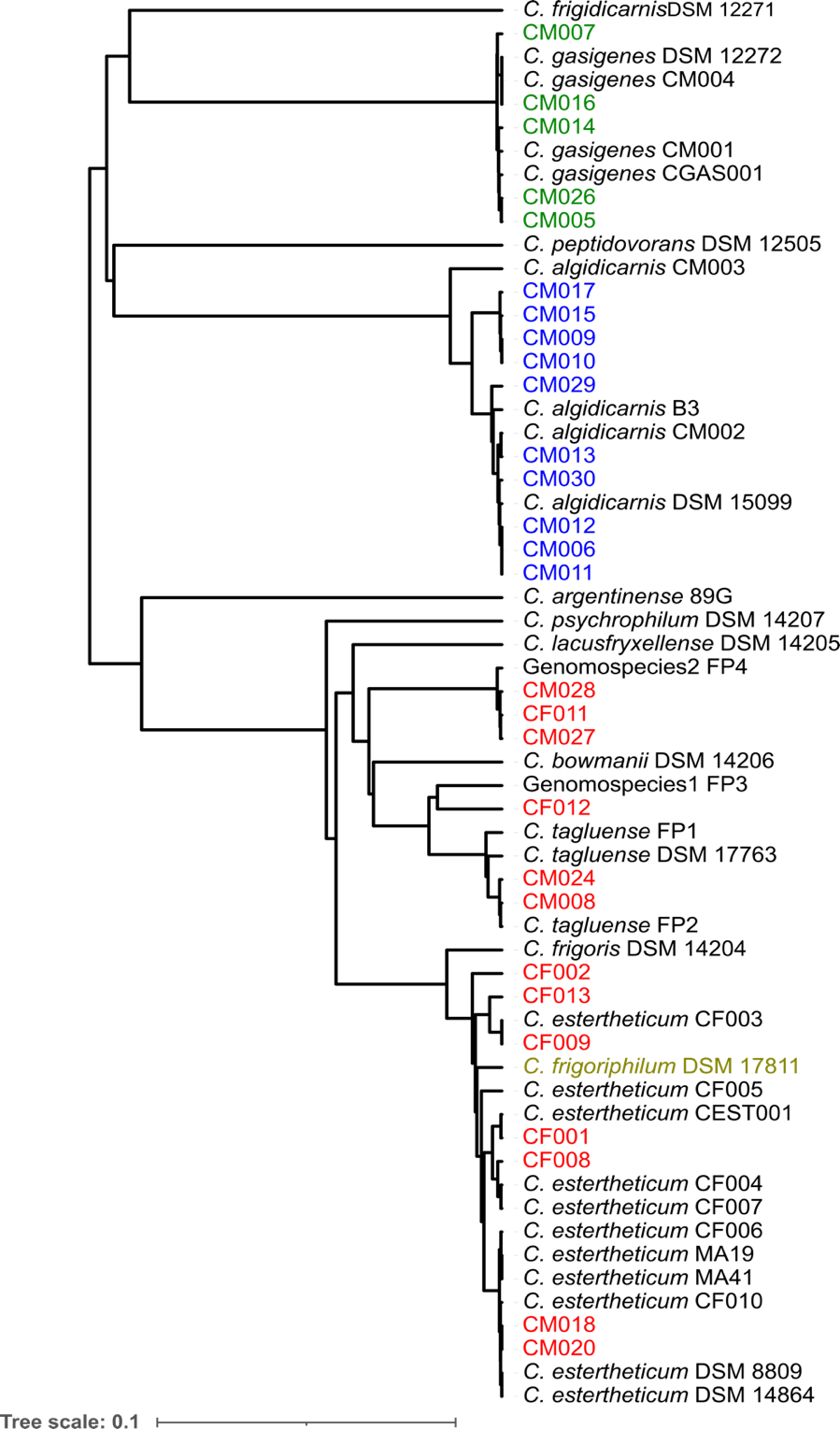


**Supplementary Figure 2.** *rpoB* gene-based phylogeny of 28 *Clostridium* spp. strains isolated from meat juice and bovine fecal samples and 32 representative clostridia strains. Thirteen strains (red) clustered within the species *Clostridium estertheticum* complex including *C. frigoriphilum* DSM 17811 (olive), which clustered within the species *C. estertheticum*. *C. algidicarnis* and *C. gasigenes* isolates from this study are highlighted in blue and green, respectively. The bar indicates 0.1 substitutions per nucleotide position.

Supplementary Table 1: Accession numbers of draft genome sequences of meat and bovine fecal sample strains isolated in the current study

| **Strain ID** | **Species** | **Sample** | **Accession number** |
| --- | --- | --- | --- |
| CF001 | *Clostridium estertheticum* | Bovine feces | GCA_019537155.1 |
| CF002 | *Clostridium estertheticum* | Bovine feces | GCA_018861295.1 |
| CF008 | *Clostridium estertheticum* | Bovine feces | GCA_019537135.1 |
| CF009 | *Clostridium estertheticum* | Bovine feces | GCA_018861325.1 |
| CF011 | *Clostridium* spp. | Bovine feces | GCA_018861595.1 |
| CF012 | *Clostridium* spp. | Bovine feces | GCA_018861755.1 |
| CF013 | *Clostridium estertheticum* | Bovine feces | GCA_018861795.1 |
| CM005 | *Clostridium gasigenes* | Beef | GCA_018861835.1 |
| CM006 | *Clostridium algidicarnis* | Lamb | GCA_018861625.1 |
| CM007 | *Clostridium gasigenes* | Lamb | GCA_018861605.1 |
| CM008 | *Clostridium tagluense* | Lamb | GCA_019537185.1 |
| CM009 | *Clostridium algidicarnis* | Lamb | GCA_018861555.1 |
| CM010 | *Clostridium algidicarnis* | Lamb | GCA_018861525.1 |
| CM011 | *Clostridium algidicarnis* | Lamb | GCA_018861495.1 |
| CM012 | *Clostridium algidicarnis* | Lamb | GCA_018861565.1 |
| CM013 | *Clostridium algidicarnis* | Lamb | GCA_018861395.1 |
| CM014 | *Clostridium gasigenes* | Lamb | GCA_018861695.1 |
| CM015 | *Clostridium algidicarnis* | Lamb | GCA_018861505.1 |
| CM016 | *Clostridium gasigenes* | Lamb | GCA_018861815.1 |
| CM017 | *Clostridium algidicarnis* | Beef | GCA_018861465.1 |
| CM018 | *Clostridium estertheticum* | Lamb | GCA_018861665.1 |
| CM020 | *Clostridium estertheticum* | Lamb | GCA_018861305.1 |
| CM024 | *Clostridium tagluense* | Beef | GCA_018861655.1 |
| CM026 | *Clostridium gasigenes* | Lamb | GCA_018861765.1 |
| CM027 | *Clostridium* spp. | Lamb | GCA_019537145.1 |
| CM028 | *Clostridium* spp. | Lamb | GCA_019537175.1 |
| CM029 | *Clostridium algidicarnis* | Lamb | GCA_018861405.1 |
| CM030 | *Clostridium algidicarnis* | Beef | GCA_018861455.1 |

Supplementary Table 2: Accession numbers of draft genome sequences of previously unsequenced *Clostridium estertheticum* complex species

| **Strain ID** | **Species** | **Sample** | **Accession number** |
| --- | --- | --- | --- |
| DSM 14204 | *Clostridium frigoris* | Microbial mat | GCA_018861865.1 |
| DSM 14205 | *Clostridium lacusfryxellense* | Microbial mat | GCA_018861735.1 |
| DSM 14206 | *Clostridium bowmanii* | Microbial mat | GCA_018861315.1 |
| DSM 14207 | *Clostridium psychrophilum* | Microbial mat | GCA_018861705.1 |
| DSM 17811 | *Clostridium* spp. | Permafrost | GCA_018861905.1 |

Supplementary Table 3: Accession numbers of 16 publicly available *Clostridium estertheticum* complex genomes used in the current study

| **Strain ID** | **Species** | **Sample** | **Accession number** |
| --- | --- | --- | --- |
| CF003 | *Clostridium estertheticum* | Bovine feces | GCA_018861435.1 |
| CF004 | *Clostridium estertheticum* | Bovine feces | GCA_019661245.1 |
| CF005 | *Clostridium estertheticum* | Bovine feces | GCA_019661205.1 |
| CF006 | *Clostridium estertheticum* | Bovine feces | GCA_018861375.1 |
| CF007 | *Clostridium estertheticum* | Bovine feces | GCA_019661225.1 |
| CF010 | *Clostridium estertheticum* | Bovine feces | GCA_018861855.1 |
| CEST001 | *Clostridium estertheticum* | Meat | GCA_013093435.1 |
| DSM 14864 | *Clostridium estertheticum* | Meat | GCA_008933175.1 |
| DSM 8809 | *Clostridium estertheticum* | Meat | GCA_001877035.1 |
| MA19 | *Clostridium estertheticum* | Meat | GCA_009295575.1 |
| MA41 | *Clostridium estertheticum* | Meat | GCA_009295545.1 |
| DSM 17763 | *Clostridium tagluense* | Permafrost | GCA_003865095.1 |
| FP1 | *Clostridium tagluense* | Meat | GCA_011065975.1 |
| FP2 | *Clostridium tagluense* | Meat | GCA_011065955.1 |
| FP3 | *Clostridium* spp. | Meat | GCA_011065935.1 |
| FP4 | *Clostridium* spp. | Meat | GCA_011065905.1 |

Supplementary Table 4: Accession numbers of 11 publicly available genomes of *Clostridium* spp. representative strain used in the current study

| **Strain ID** | **Species** | **Sample** | **Accession number** |
| --- | --- | --- | --- |
| B3 | *Clostridium algidicarnis* | Rumen | GCA_000703125.1 |
| CM002 | *Clostridium algidicarnis* | Meat | GCA_014212415.1 |
| CM003 | *Clostridium algidicarnis* | Meat | GCA_014212455.1 |
| DSM 15099 | *Clostridium algidicarnis* | Meat | GCA_002934235.1 |
| 89G | *Clostridium argentinense* | Soil | GCA_002074155.1 |
| DSM 12271 | *Clostridium frigidicarnis* | Meat | GCA_900111985.1 |
| CGAS001 | *Clostridium gasigenes* | Meat | GCA_012241495.1 |
| CM001 | *Clostridium gasigenes* | Meat | GCA_014212475.1 |
| CM004 | *Clostridium gasigenes* | Meat | GCA_014212425.1 |
| DSM 12272 | *Clostridium gasigenes* | Meat | GCA_900104115.1 |
| DSM 12505 | *Clostridium peptidovorans* | Anaerobic digester | GCA_900205925.1 |
